# Improving Text-to-SQL with a Hybrid Decoding Method

**DOI:** 10.3390/e25030513

**Published:** 2023-03-16

**Authors:** Geunyeong Jeong, Mirae Han, Seulgi Kim, Yejin Lee, Joosang Lee, Seongsik Park, Harksoo Kim

**Affiliations:** 1 Department of Artificial Intelligence, Konkuk University, 120 Neungdong-ro, Gwangjin-gu, Seoul 05029, Republic of Korea; jyjg7218@konkuk.ac.kr (G.J.);; 2 Department of Computer Science and Engineering, Konkuk University, 120 Neungdong-ro, Gwangjin-gu, Seoul 05029, Republic of Korea; 3 Division of Computer Science and Engineering & Department of Artificial Intelligence, Konkuk University, 120 Neungdong-ro, Gwangjin-gu, Seoul 05029, Republic of Korea

**Keywords:** semantic parsing, text-to-SQL, pointer network, natural language processing

## Abstract

Text-to-SQL is a task that converts natural language questions into SQL queries. Recent text-to-SQL models employ two decoding methods: sketch-based and generation-based, but each has its own shortcomings. The sketch-based method has limitations in performance as it does not reflect the relevance between SQL elements, while the generation-based method may increase inference time and cause syntactic errors. Therefore, we propose a novel decoding method, Hybrid decoder, which combines both methods. This reflects inter-SQL element information and defines elements that can be generated, enabling the generation of syntactically accurate SQL queries. Additionally, we introduce a Value prediction module for predicting values in the WHERE clause. It simplifies the decoding process and reduces the size of vocabulary by predicting values at once, regardless of the number of conditions. The results of evaluating the significance of Hybrid decoder indicate that it improves performance by effectively incorporating mutual information among SQL elements, compared to the sketch-based method. It also efficiently generates SQL queries by simplifying the decoding process in the generation-based method. In addition, we design a new evaluation measure to evaluate if it generates syntactically correct SQL queries. The result demonstrates that the proposed model generates syntactically accurate SQL queries.

## 1. Introduction

Semantic parsing is a natural language understanding task, which extracts the meaning of natural language and converts it into an executable logical form. Various tasks exist in semantic parsing, such as text-to-CFG [1], which converts natural language to context-free grammar (CFG), and text-to-code [2,3], which converts natural language into a programming language. text-to-SQL is a task that converts an unstructured natural language into a semantically corresponding structured SQL query. With the increasing accumulation of large amounts of structured text data, such as relational databases, studies in text-to-SQL have become more active in the recent years.

**Figure 1:**
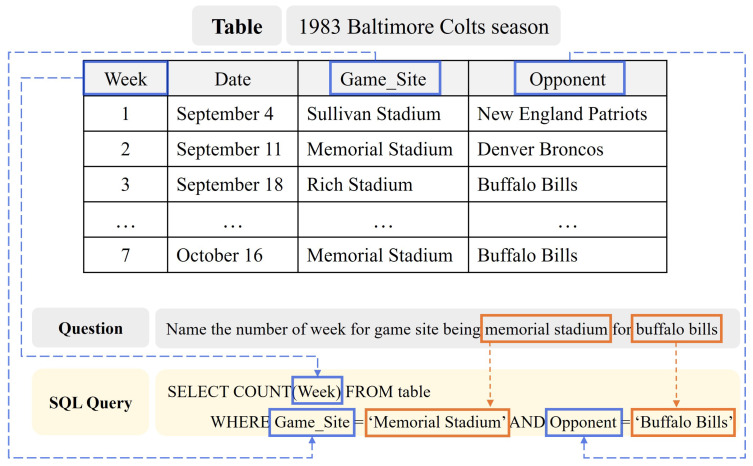
The text-to-SQL model generates an executable SQL query that corresponds to a natural language question. The referenced table schema is relevant to the natural language question. For example, the model references “Week”, “Opponent”, and “Game_Site” from the table schema and “Memorial Stadium” and “Buffalo Bills” from the natural language question to generate the SQL statement.

Figure 1 illustrates an example of a text-to-SQL task. The goal of text-to-SQL is to generate an SQL query to correctly answer a given question. For example, in Figure 1, for the question “Name the number of week for game site being memorial stadium for buffalo bills”, the text-to-SQL model generates an SQL query, SELECT COUNT(Week) FROM table WHERE Game_Site = “Memorial Stadium” AND Opponent = “Buffalo Bills”.

Text-to-SQL is highly useful in practical applications. An understanding of SQL is necessary in order to search for information in tables within a database. Therefore, it is difficult for users who lack knowledge of SQL to access and search for information in a database. However, when using the text-to-SQL model, even nontechnical users can easily search for information in a database with natural language questions by using text-to-SQL. Additionally, SQL operators (e.g., MIN, MAX, COUNT) can be used to perform calculations on numerical data, and SQL keywords (e.g., JOIN, GROUP BY) can be used to extract complex information. In summary, text-to-SQL makes it possible for nontechnical users to search databases and easily solve problems that are difficult to solve using only natural languages. Therefore, systems based on text-to-SQL are used in a variety of fields in real life, such as database management, question answering, information search, and voice assistants [4,5,6,7,8,9]. For these reasons, text-to-SQL is an important study topic in natural language processing, and various methods have been studied depending on the purpose [10,11].

Initial studies in text-to-SQL utilized rule-based methods. However, with the increasing application of deep learning in natural language processing, recent studies in text-to-SQL have used deep learning-based models. Deep learning-based text-to-SQL models consist of two parts: an encoder and a decoder. The encoder generates vector representations that encompass the natural language and structural information of the table, and the decoder utilizes the vector representations generated by the encoder to transform the input natural language into an SQL query. The decoder can be classified into two types, based on the method of generation: the sketch-based method, which generates the query by considering the components of the SQL query as slots and using a slot-filling method, and the generation-based method, which generates the query in a sequential manner.

The sketch-based method generates SQL queries based on slots, obviating the need to learn SQL syntax. While the syntactic correctness in the generated SQL query is guaranteed, multiple subtasks must be performed to output the elements of each slot. The sketch-based method has a limitation in performance due to the insufficient sharing of information among the elements when performing these subtasks.

On the other hand, the generation-based method generates SQL queries sequentially without performing subtasks for each element. This results in better performance than the sketch-based method because it predicts the next slot element by utilizing information from previously output elements. However, the generation-based method may cause syntactical errors in SQL queries as it learns the syntax of the SQL language. As a result, if the outputs of the table elements and SQL keywords are in an incorrect order, the generated SQL query may fail to be executed in the database.

**Figure 2:**
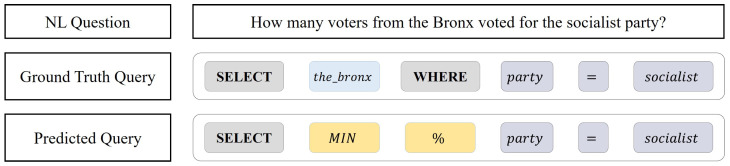
Schema of a case in which the SQL query predicted by the model contains syntactical errors. The column slot in the SELECT clause includes an aggregator, MIN, and an operator, % instead of the keyword, WHERE.

Figure 2 illustrates an instance of syntactical errors produced by the generation-based method. In the generation process, the generated SQL query may not be syntactically correct, which may result in it being unable to execute in the database.

To address the aforementioned problems, we propose a new decoding method called Hybrid decoder that combines the sketch-based method and the generation-based method. The Hybrid decoder follows a structure based on the generation-based method, generating the SQL query sequentially. It also utilizes an appropriate decoding strategy for each slot type at each step based on the sketch-based method. The proposed decoding method reflects the information of previously generated SQL elements into the current generation step; thus, it enables the model to incorporate inter-element information. It also defines the possible SQL elements that can be generated at each step, resulting in the generation of syntactically accurate SQL queries.

In addition, the proposed model uses a Value prediction module to predict the values in the WHERE clause for efficient decoding. The values in the WHERE clause are “Memorial Stadium’’ and “Buffalo Bills” in Figure 1. To extract the value candidates that appear in natural language questions, recent generation-based methods [12] use the copy mechanism to generate values in the WHERE clause. Since the copy mechanism includes tokens in the natural language question in the generation vocabulary, the size of the generation vocabulary increases. In addition, it takes a longer time to infer because it generates values for each condition. The proposed method simplifies the decoding process by predicting values simultaneously through sequence labeling [13], regardless of the number of conditions, and reduces the size of the generation vocabulary, enabling efficient SQL query generation.

Our main contributions are as follows:

- We point out the limitations of existing decoding methods, sketch-based and generation-based methods, and propose a new decoding method called Hybrid decoder, which combines the advantages of both methods and overcomes their disadvantages.
- Our proposed model achieved superior performance compared to models that applied the sketch-based method. This is because our proposed model is based on the method of sequentially generating tokens, which effectively reflects the information of the SQL elements and predicts an accurate SQL query.
- The proposed method guarantees the syntactic accuracy of the predicted SQL query. To evaluate the syntactic accuracy of the query, we designed a new evaluation measure called Syntactic Error Rate (SER). When evaluated using SER, our proposed model showed comparable performance to sketch-based methods, despite using a generation-based method.
- Our proposed method is more efficient than existing decoding methods in terms of the decoding process and vocabulary composition than existing decoding methods. It simplifies the decoding process by predicting values through sequence labeling and minimizes the size of the generation vocabulary. Consequently, our proposed method shows a faster inference speed compared to not only the generation-based method (BRIDGE [12]) but also the sketch-based method (HydraNet [14]).

The remainder of this paper is organized as follows. Section 2 describes the flow of studies in text-to-SQL, and Section 3 describes the text-to-SQL model based on the new decoding method, the Hybrid method, proposed in this paper. The dataset used for the model experiment, evaluation measures and, experimental results are described in Section 4. Finally, Section 5 concludes the paper and presents future research directions.

## 2. Related Works

Recent text-to-SQL studies differ in their datasets and decoding methods depending on the problem being solved. The most widely used datasets and decoding methods for text-to-SQL operations are as follows.

### 2.1. Dataset

The datasets for text-to-SQL tasks can be categorized into single-turn and multi-turn datasets based on the presence or absence of context. Single-turn datasets focus on generating SQL from a single natural language question, whereas multi-turn datasets consider the context of the question and conversation when generating SQL [15].

The representative datasets for the single-turn text-to-SQL study are as follows. GeoQuery is a dataset consisting of 880 natural language questions using the US geographic facts database, referred to as Geobase. Initially, it comprised 700 questions and corresponding SQL queries and a relational database schema for Geobase, as described by [16]. Subsequently, [17] annotated the remaining data for text-to-SQL tasks. Following [18], the use of 600 and 280 examples for learning and evaluation, respectively, became the standard. Scholar [17] is a dataset derived from a database of academic papers, consisting of 816 pairs of natural language questions and SQL queries. To prove that the model proposed by [17] performed well in new domains, they collected and annotated a new dataset in the academic domain and used it to evaluate their model. The data utilized in this study were generated by crowd workers and provided a database that includes information on authors, citations, journals, keywords, and dataset information of academic papers. To evaluate the model in real-world environments, [19] utilized the Microsoft Academic Search(MAS) [20], IMDB movie (https://www.imdb.com/interfaces, accessed on 30 January 2023), and Yelp (https://www.yelp.com/dataset, accessed on 30 January 2023) business review databases, and collected and published natural language questions. The datasets used in this study consist of multiple tables and the natural language questions comprise 196, 131, and 128 questions for each database, respectively. In subsequent studies, large-scale cross-domain databases have been used instead of databases for specific domains for more practical study. WikiSQL [21] is the first large-scale cross-domain text-to-SQL dataset. It consists of tables from the English Wikipedia, natural language questions corresponding to these tables, and SQL queries derived from the natural language questions. The natural language questions are user inquiries regarding a specific table, and the SQL queries are used to search the database for the answer to these questions. The queries in WikiSQL comprise only SELECT/WHERE/FROM clauses. Additionally, as the queries are for a single table, they are relatively simple and only handle a single SELECT clause and aggregations without considering the relationships between tables. The Spider dataset [22] was proposed to study a wider range of queries than the WikiSQL dataset. It comprises 200 databases from 138 different domains, including 10,181 natural language questions and 5693 SQL queries. In contrast to previous datasets that contain multiple tables within the same domain, Spider uses multiple databases and domains while incorporating complex natural language questions and SQL queries and assigns four levels of difficulty.

The representative datasets for the multi-turn text-to-SQL study are as follows. The ATIS dataset (https://www.kaggle.com/datasets/siddhadev/ms-cntk-atis, accessed on 30 January 2023) consists of 5418 utterances regarding an air reservation system, with pairs of SQL queries to answer the relational database and queries. It is a dataset composed of dialogues and is labeled with slot-filling tasks. The original dataset is not as efficient as a dataset for text-to-SQL tasks. Therefore, [17] used a dataset that has converted IN clauses to JOIN, while verifying that the query output has not been altered. CoSQL [11] is the first large-scale cross-domain conversational text-to-SQL dataset. A total of 138 domains and 200 complex databases were reconstructed from the Wizard-of-OZ (WOZ)  [23] setup, with over 3000 turns of conversation. CoSQL is composed of more than 30,000 conversations and 10,000 annotated SQL queries. Each conversation was obtained through crowd-workers who acted as users and searched the database for their answers. SQL experts transformed vague queries into clear queries, and if the user’s query was answerable in SQL, the expert constructed the data by writing the corresponding SQL and execution results. SparC [10] is a large-scale cross-domain context-dependent dataset constructed by utilizing the questions from the Spider dataset. SQL queries were annotated for each question for interrelated questions made up of conversations. CHASE [24] is a large cross-domain context-dependent Chinese dataset with 5459 interrelated questions in dialogue sequences and 17,940 natural language questions and SQL query pairs. The natural language query-SQL query pair with context is based on 280 databases, 35% of the questions are context-independent, and the difficulty of 28% of the total SQL queries is easy.

In this paper, we use a single-turn dataset, which assumes a situation in which a user asks only a single question rather than engaging in a conversation with the model.

### 2.2. Method

The initial text-to-SQL systems primarily focused on simple rule-based methods using user queries and databases [25]. Ref. [26] organized user queries into rules and designed query trees for use with databases. Ref. [27] proposed a system that enables users who lack the ability to write SQL queries to easily search information in the database using CFG-based rules. Ref. [28] used statistical parsing for the first time to convert natural language questions into SQL queries. They transformed natural language queries into logical forms using statistical parsers and mapped the logical forms to SQL queries using relational learning algorithms. Ref. [29] carried out a study using rule-based templates to directly match natural language sentences with string patterns, and used a pattern to formalize the syntax tree to match the syntax analysis tree of the natural language sentence.

However, a limitation exists in the manual design for rule-based and statistical methods, leading to the proposal of Seq2SQL [21], which applied neural networks to text-to-SQL. Seq2SQL employed an encoder-decoder neural network structure that receives a natural language question and generates an SQL query. Recent neural network-based models for text-to-SQL can be broadly classified into two categories based on their decoding methods: sketch-based and generation-based [15]. Ref. [30] first designed a sketch according to SQL grammar and then predicted and filled only the slots of the sketch using a neural network. Ref. [31] was the first to use a pre-trained language model as the encoder in text-to-SQL. The pre-trained language model encodes a natural language question, and then, the sketch-based decoder predicts an SQL query for each subtask to output the final query.

However, the problem with this approach is that all columns in the table are used as inputs to the language model, which does not consider the relationship between the natural language question and each column. To address this issue, Ref. [14] improved the encoding process by incorporating the relevance between the input natural language question and the corresponding column. In addition, the performance was improved by adding a ranking algorithm during the decoding process. The sketch-based method, which decodes pre-determined slots, is simple to process for simple queries. However, it becomes complicated when generating SQL queries that involve multiple tables or nested queries. As a result, the generation-based method is actively being studied for the generation of complex SQL queries. Ref. [32] solved the difference between natural language questions and SQL statements by adding a SemQL, an Abstract Syntax Tree, in the intermediate stage. It also used Schema Linking with word and type embeddings to understand the relationships between multiple tables. IRNet [32] attempted to find the semantic relevance between the question and schema, but it did not accurately identify the relevant schema. As a result, RAT-SQL [33] proposed a solution using self-attention to identify the exact relationship between the question and the relevant schema, while still using Schema Linking. Ref. [12] suggested using not only incorporating structure and field information from the schema but also encoding values. It also proposed using a pointer-generator network based on LSTM to decode the encoded hidden representation. This allows for the consideration of weighted words in the encoded sentence, resulting in the advantage of considering the words in the encoded sentence during the decoding process.

## 3. Methodology

**Figure 3:**
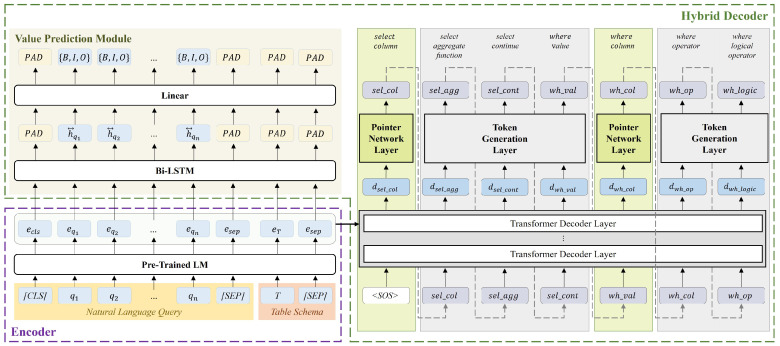
Overall architecture of the proposed model. The proposed model consists of an encoder and a decoder, and the decoder includes a subtask, the Value prediction module.

Figure 3 illustrates the overall architecture of the proposed model. The proposed model basically adopts a Seq2seq architecture, consisting of an encoder and a decoder. The encoder takes a natural language question and a table schema as input and outputs a set of vectors that reflect the interrelationship between the natural language question and the table through a language model. The decoder then takes the output vectors of the encoder as input and generates an SQL query that semantically corresponds to the natural language question.

### 3.1. Encoder

The encoder encodes the meaning of an input sequence into vector form. Pre-trained language models such as BERT [34] are used to obtain better vector representations. These pre-trained models are trained on large amounts of text corpus, enabling them to effectively understand the meaning of the input sequence. However, because of restrictions on input length, the language models may not be able to utilize all the information in a table. To alleviate this problem, BRIDGE [12] uses a method that selects the table information to be input into the encoder based on the anchor text. The anchor text refers to the cell value selected by matching the lexical similarity between the cell value of the referenced table and the natural language question. We apply the method proposed in BRIDGE and use the column names of the table and anchor text as the table schema. The natural language question and the table schema are serialized and used as input to the language model, and the vector representations that reflect the mutual relationship between the input natural language question and the table schema are obtained from the output result of the language model. The configuration of the input sequence is detailed as follows. A natural language question *Q*, which is segmented into token units, is followed by a table schema *T*, which is also segmented into token units, and the natural language question and table schema are separated by a special token [SEP]. A special token [CLS] is inserted at the beginning of the input sequence to encapsulate the overall information of the natural language question and table schema, and a special token [SEP] is appended to indicate the end of the input sequence. The table schema is inputted after a natural language question, along with additional special tokens for separating each element of the table. Special tokens are inserted before each element to distinguish between column names in the table and anchor texts. The special token [COL] is inserted before the tokenized column name *C* and used as an embedding vector for each column. Similarly, the special token [VAL] is inserted before the tokenized anchor text *V* and attached after the column name that contains the cell value. The equation used to construct the input sequence is represented as follows:

(1) $$\begin{array}{c} X=[CLS],Q,[SEP],T,[SEP] \end{array}$$

(2) $$\begin{array}{c} Q=q_{1},\ldots ,q_{n} \end{array}$$

(3) $$\begin{array}{c} T\;=\;\left[COL\right],C_{1},\left[VAL\right],V_{1},\ldots ,\left[COL\right],C_{y},\left[VAL\right],V_{z} \end{array}$$

(4) $$\begin{array}{c} C_{i}=c_{i1},\ldots ,c_{im_{i}} \end{array}$$

(5) $$\begin{array}{c} V_{j}=v_{j1},\ldots ,v_{jl_{j}} \end{array}$$

where *n* denotes the number of tokens in a natural language question *Q*, which is segmented into tokens. The *i*-th column $C_{i}$ in the table schema *T* comprises $m_{i}$ tokens, as expressed in Equation (4), and the *j*-th value $V_{j}$ in the table schema *T* comprises $l_{j}$ tokens, as expressed in Equation (5). *y*, *z* in Equation (3) represent the number of columns in the table schema *T* and the number of anchor texts, respectively.

An example of the encoder input method is as follows. The reference table for the natural language question in Figure 1 “Name the number of week for game site being memorial stadium for buffalo bills” comprises the columns “Week”, “Date”, “Opponent”, “Game_Site” (y=4). The reference table yields the anchor texts “Memorial Stadium”, “Buffalo Bills” (z=2). “Memorial Stadium” and “Buffalo Bills” are contained in the columns “Game_Site” and “Opponent”, respectively. Therefore, the table information sequence *T* for this example is as follows. T=[COL],week,[COL],date,[COL],opponent,[VAL],buffalo,bills,[COL],game,_,site,[VAL],memorial,stadium. The input token sequence *X*, which is a linear representation of a natural language question and a table schema, is encoded into a vector set $E_{emb}$ through a language model. The equation for $E_{emb}$ is as follows:

(6) $$\begin{array}{c} E_{emb}=e_{cls},e_{Q},e_{sep},e_{col},e_{c_{1}},e_{val},e_{v_{1}},\ldots ,e_{col},e_{c_{y}},e_{val},e_{v_{z}},e_{sep} \end{array}$$

(7) $$\begin{array}{c} e_{Q}=e_{q_{1}},\ldots ,e_{q_{n}} \end{array}$$

(8) $$\begin{array}{c} e_{c_{i}}=e_{c_{i1}},\ldots ,e_{c_{im_{i}}} \end{array}$$

(9) $$\begin{array}{c} e_{v_{j}}=e_{v_{j1}},\ldots ,e_{v_{j}l_{j}} \end{array}$$

In the representation $E_{emb}$, the token vectors for the special tokens [CLS], [SEP], [COL], [VAL] are denoted by $e_{cls}$, $e_{sep}$, $e_{col}$, and $e_{val}$, respectively. The token vectors for *Q*, $C_{i}$, $V_{j}$ are represented as $e_{Q}$, $e_{C_{i}}$, $e_{V_{j}}$, respectively.

### 3.2. Hybrid Decoder

The decoder uses the vectors $E_{emb}$ produced by the encoder to generate an SQL query that corresponds to the given natural language question. Hybrid decoder that we propose is a new decoding method that combines generation-based and sketch-based methods. Hybrid decoder sequentially generates an SQL query based on a generation-based structure and defines the possible SQL components that can be generated at each step based on the sketch. An appropriate decoding method is then used to generate outputs based on the corresponding slot type. The SQL components that we define in this paper are listed in Table 1. The detailed process of Hybrid decoder is expressed by Equations (10) and (11):

(10) $$\begin{array}{c} Token_{\left(j+1\right)}=\left\{\begin{array}{c} Pointer\;Network\;Layer(d_{j}),\;j=4i\;for\;i\in Z\\ Token\;Generation\;Layer(d_{j}),\;\;else \end{array}\right\}\; \end{array}$$

(11) $$\begin{array}{c} d_{j}=Transformer\;Decoder\;Block\left(E_{emb},Token_{\leq j}\right) \end{array}$$

**Figure 4:**
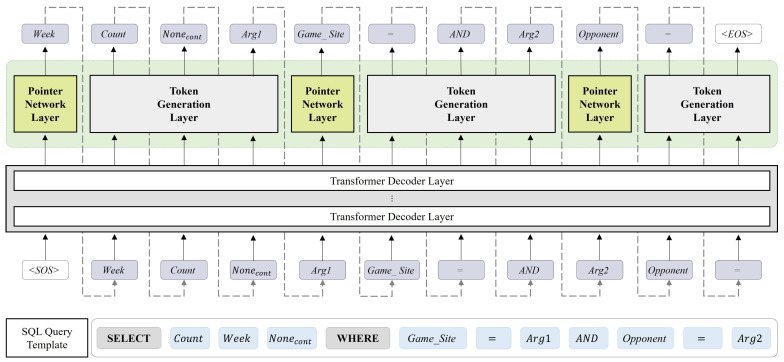
Output SQL query tokens, “Week, Count, $None_{cont}$, Arg1, Game_Site, =, AND, Arg2, Opponent, =, <EOS>”, are sorted into an executable SQL statement form. Through this sorting process, an executable SQL query “SELECT COUNT(Week) FROM table WHERE Game_Site = Arg1 AND Opponent = Arg2” can be completed.

The transformer decoder block takes the token set generated up to the *j*-th step, $Token_{\leq j}=\left\{<SOS>,Token_{1},\ldots ,Token_{j}\right\}$, and the output vector $E_{emb}$ of the encoder as input, and outputs $d_{j}$. The transformer decoder block plays an important role in determining the output at the current step by reflecting the information accumulated in the previous steps. For example, in order to predict the third-step sel_cont in the generation process, information accumulated from previous steps (Week, Count) is required, as shown in Figure 4. The decoder output $d_{j}$ that passed through the transformer decoder block generates output differently depending on the slot type. In cases where the slot type requires generating a specific column (sel_col, wh_col), a pointer network is used to select the relevant column from the input table schema. In other cases (sel_agg, sel_cont, wh_val, wh_op, wh_logic), the decoder generates the token with the highest probability from its generation vocabulary. We determine the order of decoder outputs considering the relationships between the SQL elements, based on the properties of the decoder, which play a crucial role in predicting the output of the next step from the information obtained in the previous steps. sel_col, sel_agg, sel_cont of the SELECT clause are predicted in order, and the wh_val, wh_col, wh_op, wh_logic of the WHERE clause are predicted in order as well. When generating an SQL query with a single condition, the steps corresponding to the WHERE clause are executed only once. However, if the number of conditions increases, then the steps corresponding to the WHERE clause are repeated an equivalent number of times.

Table 2 illustrates the order of the SQL-written statements and sequence of slot predictions provided by the proposed model. Since the SQL query generated by the model differs in order from the actual executable SQL query, it is not possible to execute it directly in the database. Therefore, the slot values generated by the model are sorted to form an executable SQL query format. Figure 4 shows an example of this process.

The proposed method generates Arg1, Arg2, Arg3, and Arg4 for values in the WHERE clause and predicts value candidates in the Value prediction module for efficient decoding. The final SQL query is completed by inserting appropriate cell values into Arg1, Arg2, Arg3, and Arg4, based on the lexical similarity between the value candidates and the cell values in the table. The value of the WHERE clause must be part of the natural language question *Q*; so, recent studies in sequence generation models have used the copy mechanism [35] to extract partial parts of the sequence. However, implementing the copy mechanism in the decoding phase, which predicts values by repeating at each step, increases computation and time. We apply a sequence labeling task to extract parts of the natural language question as the value of the WHERE clause. The proposed method can predict multiple value candidates at once, and by substituting the values with special tokens without considering the semantics, it can reduce the vocabulary size. In conclusion, the proposed method leverages inter-element information by incorporating previously generated SQL elements into the current generation step, based on a generation-based approach. Additionally, this method employs a sketch-based approach to define templates and generate appropriate tokens for each corresponding slot type, which ensures the generation of syntactically correct SQL queries. To improve the efficiency of the decoding process, we employ a Value prediction module for the value of the WHERE clause. This reduces the burden of generating value tokens in the decoder.

#### 3.2.1. Token Generation Layer

In the Token generation layer, the elements that compose an SQL query are generated from the generation vocabulary, using the output vectors obtained from the transformer decoder block. In all the steps of the decoder process except for the steps in which the column names are predicted, the Token generation layer is used to predict the slot values. The generation vocabulary used in the Token generation layer is listed in Table 3.

The prediction process in Token generation layer is described in Equations (12)–(14):

(12) $$\begin{array}{c} Token_{\left(j+1\right)}=argmax\left({\hat{y}}_{j}^{gn}\right)\\ where\;Token_{\left(j+1\right)}\in Vocabulary \end{array}$$

(13) $$\begin{array}{c} {\hat{y}}_{j}^{gn}=Linear\left(d_{j}\right)M_{j}^{gn} \end{array}$$

(14) $$\begin{array}{c} Linear\left(d_{j}\right)=W^{T}d_{j}+b \end{array}$$

The vector $d_{j}$ obtained from the transformer decoder block is reduced to the same size as the generation vocabulary through a linear layer and is transformed into a probability distribution over all the tokens in the generation vocabulary. In accordance with the slot type, masking is applied to transform the output vector into a probability distribution over a set of possible tokens in the generation vocabulary. *W* and *b* represent trainable parameters, weight and bias. $M_{j}^{gn}$ represents the masking matrix that restricts the generation candidates, ensuring that only valid tokens are generated according to the slot type in the (j+1)-th order. Without masking, all the tokens in the generation vocabulary have a probability of being generated regardless of the slot type, but with masking, restrictions are imposed on the token candidates that can be generated according to the slot type. Masking prevents the type errors that produce tokens that do not match the slot type and the generation of in-executable SQL queries that cause syntax errors.

**Figure 5:**
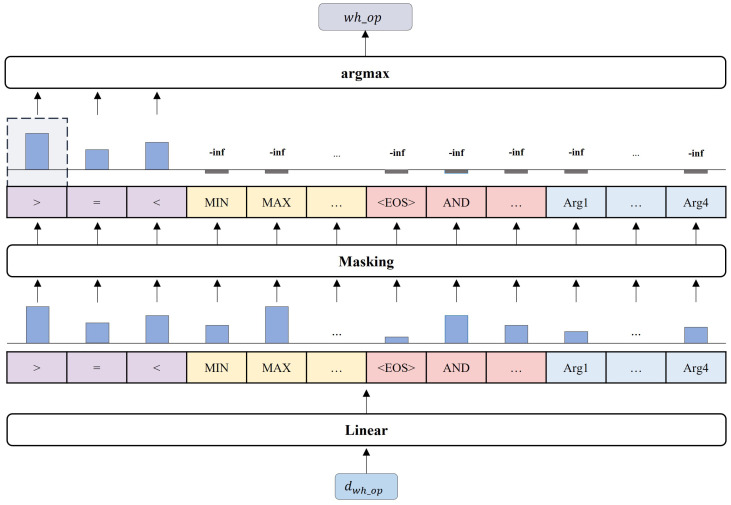
The token generation process when the step is assumed to generate wh_op. The vector $d_{wh\_op}$ generates ‘>’ through the linear layer and masking.

Figure 5 depicts the token generation process when wh_op is generated. At the wh_op step, vector $d_{wh\_op}$ from the transformer decoder is input into the Token generation layer, and a probability distribution is obtained through a linear layer. Before the application of masking, all tokens in the generation vocabulary are considered output candidates, with >, MAX, and AND being the top three candidates in the probability distribution. Although this step is to predict the aggregate function, the aggregator (MAX) and logical operator (AND) are included as output candidates. This leads to not only the possibility of incorrectly predicting the aggregate function but also the possibility of a type error that fails to predict the correct slot type. Therefore, we apply masking to the generation probability distribution, and eliminate the probability of generating tokens other than the slot type to be generated in the current step. After masking is applied, only the tokens in the generation vocabulary corresponding to the operator, such as >, =, <, are considered as output candidates, and the output token is generated accordingly.

#### 3.2.2. Pointer Network Layer

The Pointer network layer selects an appropriate column through an attention operation between the output vector of the transformer decoder block and a given table schema. The Pointer network layer is employed in all the steps for column prediction (sel_col, wh_col) to predict the slot value. The equation is as follows:

(15) $$\begin{array}{c} {\hat{y}}_{j}^{pn}=Pointer\;Network\left(d_{j},E_{emb}\right) \end{array}$$

(16) $$\begin{array}{c} c=argmax({\hat{y}}_{j}^{pn}M_{j}^{pn}) \end{array}$$

The current decoder hidden vector $d_{j}$ and the output vector of the encoder $E_{emb}$ are input into the pointer network and converted into a ${\hat{y}}_{j}^{pn}$. Since the pointer network is used for column selection, we mask the attention scores to distinguish between columns and non-columns, limiting the selection to only within columns. $M_{j}^{pn}$ represents a masking matrix that restricts the candidates to columns that can be selected using the (j+1)-th slot type. The column *c* with the highest probability distribution, determined by argmax, is selected among the candidate columns. Attention mechanism is utilized to evaluate the mutual correlation between the arguments (Query, Key) involved in the operation. In the proposed model, the current decoder hidden vector $d_{j}$ and the output vector of the encoder $E_{emb}$ are set as Query and Key in the attention operation to determine the mutual association. The higher the attention score, the higher the mutual correlation is perceived, and the column with the highest attention score is selected as the output for the current step. As pointer the network mechanism cannot select multiple tokens in a single step, the model is trained to select the special token [COL] in front of each column name. The use of a Pointer network layer allows for the accurate prediction of the column in a table schema that is most relevant to the current decoder step token $d_{j}$, even if the number of columns in the table schema increases. This enables the model to adapt to a table schema with variable lengths. Additionally, the need to include the names of all columns in the generation vocabulary is eliminated, reducing the size of the vocabulary and preventing the occurrence of grammatical errors in column names because it is selected from the given table schema. The attention mechanism used in the proposed model to perform the Pointer network layer is the scaled-dot product attention, and its equation is as follows:

(17) $$\begin{array}{c} Attention\;Score\left(Query,Key\right)=\frac{Query\cdot Key^{T}}{\sqrt{dim_{h}}} \end{array}$$

Query and Key correspond to the decoder hidden vector $d_{j}$ at current step j and the output vectors from the encoder $E_{emb}$, respectively. $dim_{h}$ represents the size of the hidden vector. In the Pointer network layer, the attention score between the language model’s output vector $E_{emb}$ and the decoder’s hidden vector $d_{j}$ at the current step is used to predict the column of the SQL query. The pointer network is only executed in the steps for predicting the columns of the SELECT and the WHERE clauses, so only $d_{sel\_col}$ and $d_{wh\_col}$ among the decoder hidden vectors are used in the attention mechanism. In the step of predicting the column name in the SELECT clause, the attention score between the first step of the decoder, denoted by $d_{sel\_col}$, and the output sequence of the encoder is calculated, while in the step of predicting the column name in the WHERE clause, the attention score between the output vector of the previous step, denoted by $d_{wh\_col}$, and the output sequence of the encoder is calculated. After performing the attention mechanism, a mask is applied to the scores of the tokens excluding the [COL] tokens, and only the attention scores corresponding to the [COL] token are used. Only the tokens corresponding to the columns from the table schema can be output by applying a column mask that selects the special token [COL] inserted before the column.

**Figure 6:**
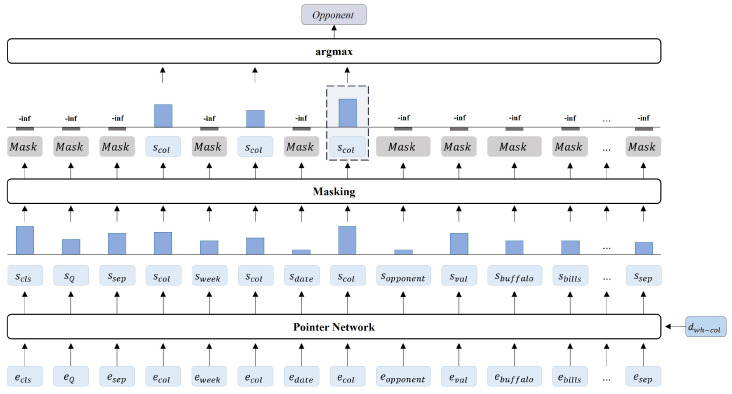
Process of pointer network assuming the step for predicting $d_{wh\_col}$. The vector $d_{wh\_col}$ generates “Opponent” through the linear layer and masking.

Figure 6 illustrates the column prediction process. The decoder output $d_{wh\_col}$ from the previous step is used as an input in the current step, and the attention operation between $d_{wh\_col}$ and the output vector of the encoder $E_{emb}$ are performed in the Pointer network layer. Tokens that are relevant to $d_{wh\_col}$ are produced by the attention score, which is the result of the Pointer network layer. Tokens that are considered highly relevant to $d_{wh\_col}$ include the tokens which are not columns, such as “buffalo” and “bills”. This implies that non-column tokens may be predicted in the decoding steps when columns are intended to be predicted. Therefore, we apply a column mask to the attention scores to exclude non-column vectors from the candidates. After masking, constraints are imposed on the selection candidates by only considering the attention scores of the column special tokens [COL], $s_{col}$, so the most relevant column, “Opponent”, is selected.

#### 3.2.3. Value Prediction Module

The proposed method performs a subtask to predict the WHERE values in an input sentence. The Value prediction module determines value candidates for the WHERE clause from natural language questions through sequence labeling. Previous studies predicted values in WHERE clauses using a copy mechanism or span prediction. However, the copy mechanism uses attention to copy a specific part of the input sequence as the output of the decoder, resulting in an expanded generation vocabulary and longer inference time due to the repeated generation of tokens for each condition. Additionally, span prediction requires a span sorting process based on the start and end scores in the natural language question. By contrast, the Value prediction module predicts the values of the WHERE clause using a sequence labeling model that assigns a label to each token in the input sequence. As a result, it can predict all values at once even if the number of conditions increases and avoid unnecessary sorting processes, rendering it more efficient in terms of speed compared to the previous methods. The Value prediction module uses sequence labeling to label each token in a natural language question with BIO tags to identify the cell values present in the question. Tag B represents the token that starts the cell value, I represents the tokens that correspond to the cell value but are not the starting tokens, and O represents the tokens that are not cell values. The detailed process for predicting the cell values is as follows. First, the cell values used in the WHERE clause are part of the input natural language question, so only the natural language question embedding vector $e_{Q}$ is used, excluding the part corresponding to the table schema among the output vector $E_{emb}$ from the language model. $e_{Q}$ passes through a bidirectional LSTM to incorporate contextual information. The equation is as follows:

(18) $$\begin{array}{c} {\vec{h}}_{i}=LSTM\left(e_{Q},{\vec{h}}_{i-1}\right) \end{array}$$

(19) $$\begin{array}{c} {\overset{\leftarrow }{h}}_{i}=LSTM\left(e_{Q},{\overset{\leftarrow }{h}}_{i-1}\right) \end{array}$$

(20) $$\begin{array}{c} {\overset{↔}{h}}_{i}=\left[{\vec{h}}_{i};{\overset{\leftarrow }{h}}_{i}\right] \end{array}$$

(21) $$\begin{array}{c} H={\overset{↔}{h}}_{q_{1}},{\overset{↔}{h}}_{q_{2}},{\overset{↔}{h}}_{q_{3}},\ldots ,{\overset{↔}{h}}_{q_{n}} \end{array}$$

The set of vectors *H* that have passed through the LSTM is passed through a linear layer to attach B, I, and O tags to each token of the natural language question. The equation is as follows:

(22) $$\begin{array}{c} {\hat{y}}^{vpm}=W^{T}H+b \end{array}$$

The linear layer receiving *H* has the trainable parameters *W* and *b*. Based on the tag information attached to each token, it is possible to predict the value of a WHERE clause in a natural language query. Finally, it is necessary to substitute the value candidates of the WHERE clause predicted in the Value prediction module with the slots of the SQL templates, Arg1, Arg2, Arg3, and Arg4. The proposed method uses a lexical-based similarity score to connect the predicted value candidate with the most similar cell value in the table by selecting the cell value from among those that can be substituted. The cell values of the table are targeted at replaceable cell values rather than at all cell values. For example, if the generated result is assumed to be WHERE col1=Arg1, the value that can be placed in Arg1 must be selected from the cell values included in col1.

**Figure 7:**
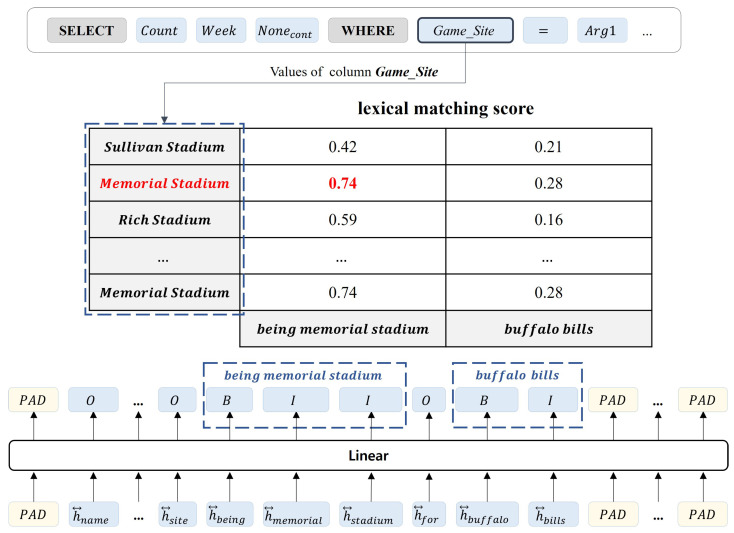
Example of the process of obtaining candidate values through the Value prediction module and substituting them with cell values in the table. Arg1 is substituted based on the candidate values predicted from the Value prediction module.

Figure 7 is an example of the process of obtaining value candidates and replacing them with values in the WHERE clause through the Value prediction module. With the BIO results attached to each natural language token, “being memorial stadium” and “buffalo bills” can be obtained as value candidates. The generated SQL query template is SELECT COUNT(Week) FROM table WHERE Game_Site = Arg1 AND Opponent = Arg2, so Arg1 and Arg2 must be replaced with appropriate value candidates. The process of restoring Arg1 involves using the information of the column “Game_Site” to calculate the lexical similarity score between all candidate values and the cell values contained in the “Game_Site” column of the table. The cell value and candidate value with the highest lexical similarity are “Memorial Stadium” and “being Memorial Stadium”, so Arg1 is finally replaced with “Memorial Stadium”. If this process is repeated for each condition, the final executable SQL statement, SELECT COUNT(Week) FROM table WHERE Game_Site = “memorial stadium” AND Opponent = “buffalo bills”, can be obtained. The advantages of the proposed method are that even if the value to be substituted in the table cannot be accurately predicted from the natural language question during the generation process, the highest similarity can be obtained in the lexical-based similarity matching process, which prevents errors in sequence labeling. In Figure 7, the cell value to be substituted in the table is “Memorial Stadium”, but even if it is predicted as “being memorial stadium”, it can be restored to the correct value. Furthermore, it allows more efficient generation of executable SQL queries. If the predicted value in the natural language question is not included in the table as a synonym, obtaining accurate answers is difficult. However, by using the proposed method, the value can be directly obtained from the table cell; so, this problem can be solved and more accurate results can be obtained when executing SQL queries.

### 3.3. Training

The cross-entropy loss function was used for training. The formula for cross-entropy is as follows:

(23) $$\begin{array}{c} Loss_{ce}=-\frac{1}{N}\sum_{i=1}^{N}\sum_{j\in Class}^{C}{\hat{y}}_{ij}log\left(y_{ij}\right) \end{array}$$

The final loss function Loss used for training consists of the loss function values $Loss_{vpm}$, $Loss_{gn}$, and $Loss_{pn}$ generated in the sequence labeling task of the Value prediction module, token generation, and pointer network, respectively. The formula for the final loss function Loss of the proposal model is as follows:

(24) $$\begin{array}{c} Loss=Loss_{vpm}+Loss_{gn}+Loss_{pn} \end{array}$$

## 4. Experiments

### 4.1. Metric

We use Logical Form (LF) as a metric for evaluating the performance of the proposed model. LF is also referred to as the Exact Set Match Accuracy (EM), which is calculated by comparing the predicted SQL query with the ground-truth SQL query. The equation is as follows:

(25) $$\begin{array}{c} Score_{LF}\left(\hat{Y},\;Y\right)=\left\{\begin{array}{c} 1,\;\hat{Y}=Y\\ 0,\;\hat{Y}\neq Y \end{array}\right\}\;\\ where\;\hat{Y}=\left\{\left(\hat{k^{i}},\hat{v^{i}}\right)|i\in \left(1,m\right)\right\},\;Y=\left\{\left(k^{i},v^{i}\right)|i\in \left(1,m\right)\right\} \end{array}$$

(26) $$\begin{array}{c} LF=\frac{1}{N}\sum_{n=1}^{N}Score_{LF}\left(\hat{Y_{n}},\;Y_{n}\right) \end{array}$$

*N* denotes the total number of data samples. $Score_{LF}\left(\hat{Y},Y\right)$ assigns a score of one if the ground-truth SQL query *Y* and the predicted SQL query $\hat{Y}$ are identical ($Y=\hat{Y}$), and a score of zero if they are not ($Y\neq \hat{Y}$). $\hat{Y}$ and *Y* represent the sets of the predicted and ground-truth SQL queries, respectively. LF is the average of $Score_{LF}$($\hat{Y},Y$) calculated over all data samples. As a natural language question can have multiple corresponding SQL queries, LF is a strict metric that evaluates the ability of the model to generate semantically equivalent SQL queries. Execution Accuracy (EX) is calculated by comparing the execution results of the ground-truth SQL query and the predicted SQL query. The equations for $Score_{EX}\left(\hat{V},V\right)$ and EX are as follows:

(27) $$\begin{array}{c} Score_{EX}\left(\hat{V},\;V\right)=\left\{\begin{array}{c} 1,\;\hat{V}=V\\ 0,\;\hat{V}\neq V \end{array}\right\}\; \end{array}$$

(28) $$\begin{array}{c} EX=\frac{1}{N}\sum_{n=1}^{N}Score_{EX}\left(\hat{V_{n}},\;V_{n}\right) \end{array}$$

$Score_{EX}\left(\hat{V},V\right)$ assigns a score of one if the execution result *V* of the ground-truth SQL query *Y* and the execution result $\hat{V}$ of the predicted SQL query $\hat{Y}$ are identical, and a score of zero if they are not ($V\neq \hat{V}$). EX represents the average of $Score_{EX}\left(\hat{V},V\right)$ calculated over all data samples.

### 4.2. Dataset

The WikiSQL dataset was used for experimentation and evaluation. WikiSQL is a dataset that is widely used for single-turn text-to-SQL tasks. The WikiSQL dataset consists of 80,654 natural language questions and 24,241 tables extracted from Wikipedia, of which 56,355 are used as training data, 8421 as development data, and 15,878 as evaluation data. A single natural language question may have multiple corresponding SQL queries, and each SQL statement contains a SELECT clause with a maximum of one aggregate operator and a WHERE clause with a maximum of four conditions joined by an operator AND.

### 4.3. Experimental Parameters and Environment

We use the BERT-large-uncased model as the language model. The specific hyperparameters used in training are listed in Table 4, and the experimental environment is listed in Table 5.

### 4.4. Comparison of Overall Performance

We conduct a performance evaluation of the proposed method by comparing its results with those of existing models to determine whether it generates SQL queries that correspond semantically to natural language questions. The comparison was performed using SQLova, X-SQL, HydraNet (sketch-based models), and BRIDGE (a generation-based model). The evaluation metrics used are LF and EX, and the results are listed in Table 6. Additionally, to verify the time efficiency of the proposed method for generating SQL queries, we measure the inference speed of the models. For a fair comparison, we measure the inference time in the same environment. To measure the inference time per sentence, the batch size was set to 1, and the average inference time was calculated based on three separate measurements.

The experimental results show that LF and EX performances of the proposed model improved to 83.5 and 89.1, respectively, compared to sketch-based models (SQLova, X-SQL, and HydraNet). The proposed model applies a generation-based structure and arranges the order by considering the mutual information between slots when generating the SQL elements, resulting in an effective reflection of the mutual information between the SQL elements. In addition, the proposed method shows significant results in terms of time efficiency, with an inference speed of approximately 71.5 ms/sentence, which is faster than both generation-based BRIDGE and sketch-based HydraNet. Therefore, based on these experiment results, Hybrid decoder outperforms sketch-based methods by effectively reflecting the mutual information between SQL elements and efficiently generates SQL queries by simplifying the decoding process in generation-based methods.

### 4.5. Comparison of Performance by Each SQL Element

We measured the performance of each component that constitutes an SQL query and compared its performance with that of existing models. The models used for comparison are SQLova, X-SQL, and HydraNet, which are sketch-based models. The target elements of the experiment are the subtasks performed in the sketch-based method, sel_col, sel_agg, wh_num, wh_col, wh_op, and wh_val. The test set is used for experiments, and the results are presented in Table 7.

The results of the experiment show that the proposed model exhibits similar performance to other sketch-based models in terms of sel_col, sel_agg, and wh_col, but it outperforms the comparison models in terms of wh_num, wh_op, and wh_val. The comparison models use a span-prediction task to find the start and end positions in the natural language question to predict the value in the WHERE clause. However, we apply a sequence labeling model to predict the value from the natural language question. Table 8 lists the performance of labeling BIO tags, which is the result of the Value prediction module.

### 4.6. Comparison of Syntactic Error

We compare the syntactic error rate of the proposed model with those of the comparison models to verify whether the proposed model generates syntactically correct SQL queries. To compare syntactic error rates, we design a new evaluation metric, Syntactic Error Rate (SER), to evaluate the syntactic accuracy of SQL queries:

(29) $$SER=100\times \frac{1}{N}\sum_{n=1}^{N}Score_{SER}\left(\hat{Y_{n}}\right)$$

*N* denotes the total number of data samples. The score $Score_{SER}\left(\hat{Y_{n}}\right)$ is assigned a value of zero if the predicted SQL query $\hat{Y}$ is free of syntactic errors and is executable, or one if it contains syntactic errors and an error occurs during execution. SER is calculated as the average of $Score_{SER}\left(\hat{Y}\right)$ over all data samples. Syntactic errors are determined by executing SQL queries in a database. We consider SQLova and HydraNet as comparison models, both of which have publicly available results for the SQL queries generated by the model. Both models use a sketch-based method; thus, if the proposed method produces a similar SER score, then it can be considered a syntactically accurate generation method.

Table 9 lists the results of the comparison experiment for the syntactic errors of the models on the WikiSQL evaluation set. The sketch-based method generates SQL queries based on slots, ensuring the syntactic accuracy of the transformed SQL query. Therefore, SQLova and HydraNet models show low error rates of approximately 0.14% and 0.12%, respectively. Most errors occur as a result of a mismatch between the data type of the column and the value. In other words, the value is inconsistent with the type of data assigned to the column (e.g., when the column type is real and the value is of string type). The proposed model shows an SER score of zero, demonstrating that the hybrid decoding method selectively performs the pointer network and generation on the type of slots and uses an appropriate method for each element type. This guarantees the syntactic accuracy of the generated SQL query.

## 5. Conclusions

We address the limitations of the existing decoding methods, sketch-based, and generation-based approaches and propose a new decoding method, Hybrid decoder, which combines their respective advantages.

Hybrid decoder follows a generation-based structure and generates SQL queries sequentially. At each step, a token is generated based on the slot type of the corresponding step, using an appropriate decoding method, with the sketch as the basis. This enables the model to effectively reflect the inter-element information of SQL elements, as it incorporates information from previously generated SQL elements into the current generation step. Furthermore, it defines possible SQL elements that can be generated at each step based on the sketch, allowing for the generation of syntactically correct SQL queries.

Additionally, we introduce a Value prediction module, a subtask for predicting the values in the WHERE clause. Previous models used copy mechanism or span prediction to predict values, which has the drawback of taking a long inference time. However, the Value prediction module can simplify the decoding process and reduce the size of the generation vocabulary by simultaneously predicting values through sequence labeling, enabling efficient SQL statement generation regardless of the number of conditions.

The results of evaluating the significance of the proposed method through the experiments are as follows. First, the proposed model outperforms sketch-based models, as the hybrid decoding method based on generation effectively reflects the mutual information of the SQL elements, leading to improved performance. Second, we design a new evaluation measure, SER, to evaluate whether the model generates syntactically accurate SQL queries. Despite using the generation-based method, the performance of the proposed model is similar to that of the sketch-based method, demonstrating that the proposed model generates syntactically accurate SQL queries. Finally, we evaluate the inference speed to verify the time efficiency of the proposed method in generating SQL queries. As a result, the proposed decoding method demonstrates a faster model inference speed than BRIDGE, which is a generation-based method, and also faster than HydraNet, which is a sketch-based method.

Hybrid decoder employs a combination of sketch-based and generation-based methods, which enables the integration of the attributes and algorithms utilized in both methods. For future work, we plan to improve the performance through the blended utilization of prior methods.

## Figures and Tables

**Table 1:** SQL elements and their descriptions.

Terms	Abbreviations	Description
sel_col	select-column	column of SELECT clause
sel_agg	select-aggregate function	aggregate function of SELECT clause
sel_cont	select-continue	Indicates whether an SQL syntax continues, e.g., [EOS] denotes the termination of the SQL, and None_cont indicates the continuation of the SQL and the start of the WHERE clause.
wh_col	where-column	column of WHERE clause
wh_op	where-operator	comparison operator of WHERE clause
wh_logic	where-logical operator	logical operator of WHERE clause
wh_num	where-number	condition number of WHERE clause
wh_val	where-value	value of WHERE clause

**Table 2:** Comparison of SQL generation order and written order.

**Generation Order**	sel_col→ sel_agg→ sel_cont→ wh_val→ wh_col→ wh_op→ wh_logic
**Written Order**	sel_agg→ sel_col→ sel_cont→ wh_col→ wh_op→ wh_val→ wh_logic

**Table 3:** Types and description of tokens in generation vocabulary used in Token generation layer.

Group	Token	Description
operator	=, >, <	tokens that indicate operators
aggregate function	$None_{agg}$, MAX, MIN, COUNT, SUM, AVG	tokens that indicate aggregate function
logical operator	AND, $None_{cont}$	tokens that indicate the continuation of where condition
value of where condition	Arg1, Arg2, Arg3, Arg4	tokens that indicate the value of where condition
else	[PAD], [SOS], [EOS]	tokens that are not directly included in SQL statement, but used as a tool in the generation process

**Table 4:** Experimental parameter settings.

Parameter Type	Parameter Value
batch size	128
learning rate	0.00005
dropout	0.3
epoch	30
number of transformer decoder layer	8
number of heads for attention head in the decoder layer	8
size of the vector of head for attention head in decoder layer	128

**Table 5:** Experimental environment settings.

Object	Environment
system	Ubuntu 18.04.6 LTS
GPU	NVIDIA RTX 8000
Python version	Python 3.8.15
Pytorch	1.13.1
transformers library	4.25.1
CUDA version	11.6

**Table 6:** Accuracy (LF, EX) of SQL query generation and inference speed (ms/sentence) on the WikiSQL dataset.

Model	Base Model	Decoding Method	Test (LF)	Text (EX)	Inference Time (ms/Sentence)
SQLova	Bert-Large	sketch-based	80.7	86.2	41.1
X-SQL	MT-DNN	sketch-based	83.3	88.7	-
HydraNet	Bert-Large	sketch-based	83.4	88.6	85.2
BRIDGE	Bert-Large	generation-based	85.7	91.1	124.6
Ours	Bert-Large	hybrid	83.5	89.1	71.5

**Table 7:** Comparison of partial performance of the model.

Model	Base Model	Decoding Method	sel_col	sel_agg	wh_num	wh_col	wh_op	wh_val
SQLova	Bert-Large	sketch-based	96.8	90.6	98.5	94.3	97.3	95.4
X-SQL	MT-DNN	sketch-based	97.2	91.1	98.6	95.4	97.6	96.6
HydraNet	Bert-Large	sketch-based	97.6	91.4	98.4	95.4	97.4	96.1
Ours	Bert-Large	hybrid	97.2	91.0	99.3	94.0	98.4	97.3

**Table 8:** Sequence labeling performance of Value prediction module.

Group	Precision	Recall	F1-Score	Tag Count
B	98	99	99	21,337
I	100	98	99	39,001
O	100	100	100	177,605
Macro average	99	99	99	237,943

**Table 9:** Comparison of syntax error on Syntactic Error Rate (SER).

Model	Decoding Method	SER (%)
SQLova	sketch-based	0.14
HydraNet	sketch-based	0.12
Ours	hybrid	0.00

## Data Availability

The data utilized in this study are publicly available at https://github.com/salesforce/WikiSQL (accessed on 30 January 2023).

## References

[1] Luz F.F., Finger M. (2018). Semantic Parsing: Syntactic assurance to target sentence using LSTM Encoder CFG-Decoder. arXiv.

[2] Soliman A.S., Hadhoud M.M., Shaheen S.I. (2022). MarianCG: A code generation transformer model inspired by machine translation. J. Eng. Appl. Sci..

[3] Yin P., Neubig G. A Syntactic Neural Model for General-Purpose Code Generation. Proceedings of the 55th Annual Meeting of the Association for Computational Linguistics (Volume 1: Long Papers).

[4] Hristidis V., Papakonstantinou Y., Gravano L. (2003). Efficient IR-style keyword search over relational databases. Proceedings of the 2003 VLDB Conference.

[5] Hristidis V., Papakonstantinou Y. (2002). Discover: Keyword search in relational databases. Proceedings of the VLDB’02: Proceedings of the 28th International Conference on Very Large Databases.

[6] Luo Y., Lin X., Wang W., Zhou X. Spark: Top-k keyword query in relational databases. Proceedings of the 2007 ACM SIGMOD International Conference on Management of Data.

[7] Zhong Z., Lee M.L., Ling T.W. (2015). Answering Keyword Queries involving Aggregates and Group-Bys in Relational Databases. Technical Report. https://dl.comp.nus.edu.sg/bitstream/handle/1900.100/5163/TRA7-15.pdf?sequence=2&isAllowed=y.

[8] Popescu A.M., Armanasu A., Etzioni O., Ko D., Yates A. Modern natural language interfaces to databases: Composing statistical parsing with semantic tractability. Proceedings of the COLING 2004: Proceedings of the 20th International Conference on Computational Linguistics.

[9] Kamath A., Das R. (2018). A survey on semantic parsing. arXiv.

[10] Yu T., Zhang R., Yasunaga M., Tan Y.C., Lin X.V., Li S., Er H., Li I., Pang B., Chen T. SParC: Cross-Domain Semantic Parsing in Context. Proceedings of the 57th Annual Meeting of the Association for Computational Linguistics.

[11] Yu T., Zhang R., Er H., Li S., Xue E., Pang B., Lin X.V., Tan Y.C., Shi T., Li Z. CoSQL: A Conversational Text-to-SQL Challenge Towards Cross-Domain Natural Language Interfaces to Databases. Proceedings of the 2019 Conference on Empirical Methods in Natural Language Processing and the 9th International Joint Conference on Natural Language Processing (EMNLP-IJCNLP).

[12] Lin X.V., Socher R., Xiong C. Bridging Textual and Tabular Data for Cross-Domain Text-to-SQL Semantic Parsing. Proceedings of the Findings of the Association for Computational Linguistics: EMNLP 2020.

[13] Kim H., Kim H. (2021). Fine-grained named entity recognition using a multi-stacked feature fusion and dual-stacked output in Korean. Appl. Sci..

[14] Lyu Q., Chakrabarti K., Hathi S., Kundu S., Zhang J., Chen Z. (2020). Hybrid Ranking Network for Text-to-SQL. arXiv.

[15] Qin B., Hui B., Wang L., Yang M., Li J., Li B., Geng R., Cao R., Sun J., Si L. (2022). A Survey on Text-to-SQL Parsing: Concepts, Methods, and Future Directions. arXiv.

[16] Popescu A.M., Etzioni O., Kautz H. (2003). Towards a Theory of Natural Language Interfaces to Databases. Proceedings of the 8th International Conference on Intelligent User Interfaces, IUI ’03.

[17] Iyer S., Konstas I., Cheung A., Krishnamurthy J., Zettlemoyer L. Learning a Neural Semantic Parser from User Feedback. Proceedings of the 55th Annual Meeting of the Association for Computational Linguistics (Volume 1: Long Papers).

[18] Zettlemoyer L.S., Collins M. (2005). Learning to Map Sentences to Logical Form: Structured Classification with Probabilistic Categorial Grammars. Proceedings of the Twenty-First Conference on Uncertainty in Artificial Intelligence, UAI’05.

[19] Yaghmazadeh N., Wang Y., Dillig I., Dillig T. (2017). SQLizer: Query Synthesis from Natural Language. Proc. ACM Program. Lang..

[20] Sinha A., Shen Z., Song Y., Ma H., Eide D., Hsu B.J.P., Wang K. (2015). An Overview of Microsoft Academic Service (MAS) and Applications. Proceedings of the 24th International Conference on World Wide Web, WWW ’15 Companion.

[21] Zhong V., Xiong C., Socher R. (2017). Seq2SQL: Generating Structured Queries from Natural Language using Reinforcement Learning. arXiv.

[22] Yu T., Zhang R., Yang K., Yasunaga M., Wang D., Li Z., Ma J., Li I., Yao Q., Roman S. Spider: A Large-Scale Human-Labeled Dataset for Complex and Cross-Domain Semantic Parsing and Text-to-SQL Task. Proceedings of the 2018 Conference on Empirical Methods in Natural Language Processing.

[23] Mrkšić N., Ó Séaghdha D., Wen T.H., Thomson B., Young S. Neural Belief Tracker: Data-Driven Dialogue State Tracking. Proceedings of the 55th Annual Meeting of the Association for Computational Linguistics (Volume 1: Long Papers).

[24] Guo J., Si Z., Wang Y., Liu Q., Fan M., Lou J.G., Yang Z., Liu T. Chase: A Large-Scale and Pragmatic Chinese Dataset for Cross-Database Context-Dependent Text-to-SQL. Proceedings of the 59th Annual Meeting of the Association for Computational Linguistics and the 11th International Joint Conference on Natural Language Processing (Volume 1: Long Papers).

[25] Deng N., Chen Y., Zhang Y. Recent Advances in Text-to-SQL: A Survey of What We Have and What We Expect. Proceedings of the 29th International Conference on Computational Linguistics, International Committee on Computational Linguistics.

[26] Li F., Jagadish H.V. (2014). Constructing an Interactive Natural Language Interface for Relational Databases. Proc. VLDB Endow..

[27] Mahmud T., Azharul Hasan K.M., Ahmed M., Chak T.H.C. A rule based approach for NLP based query processing. Proceedings of the 2015 2nd International Conference on Electrical Information and Communication Technologies (EICT).

[28] Tang L.R., Mooney R.J. (2000). Automated Construction of Database Interfaces: Integrating Statistical and Relational Learning for Semantic Parsing. Proceedings of the 2000 Joint SIGDAT Conference on Empirical Methods in Natural Language Processing and Very Large Corpora: Held in Conjunction with the 38th Annual Meeting of the Association for Computational Linguistics—Volume 13, EMNLP ’00.

[29] Kate R.J., Wong Y.W., Mooney R.J. (2005). Learning to Transform Natural to Formal Languages. Proceedings of the 20th National Conference on Artificial Intelligence—Volume 3, AAAI’05.

[30] Xu X., Liu C., Song D. (2018). SQLNet: Generating Structured Queries From Natural Language without Reinforcement Learning. arXiv.

[31] Hwang W., Yim J., Park S., Seo M. (2019). A Comprehensive Exploration on WikiSQL with Table-Aware Word Contextualization. arXiv.

[32] Guo T., Gao H. (2019). Content Enhanced BERT-based Text-to-SQL Generation. arXiv.

[33] Wang B., Shin R., Liu X., Polozov O., Richardson M. RAT-SQL: Relation-Aware Schema Encoding and Linking for Text-to-SQL Parsers. Proceedings of the 58th Annual Meeting of the Association for Computational Linguistics.

[34] Devlin J., Chang M.W., Lee K., Toutanova K. BERT: Pre-training of Deep Bidirectional Transformers for Language Understanding. Proceedings of the 2019 Conference of the North American Chapter of the Association for Computational Linguistics: Human Language Technologies, Volume 1 (Long and Short Papers).

[35] Gu J., Lu Z., Li H., Li V.O. Incorporating Copying Mechanism in Sequence-to-Sequence Learning. Proceedings of the 54th Annual Meeting of the Association for Computational Linguistics (Volume 1: Long Papers).

